# Eosinophilic enteritis accompanied by cytomegalovirus disease: a case report

**DOI:** 10.1186/s12876-022-02274-1

**Published:** 2022-04-28

**Authors:** Yuichi Yamaga, Masahiro Mizuno, Shunji Okae, Mikako Nio-Tamaoki, Kenji Masuo, Yoko Mashimo-Matsuo, Junya Tanaka, Motoshige Nabeshima

**Affiliations:** 1 Department of Gastroenterology, Mitsubishi Kyoto Hospital, Kyoto, Japan; 2 Department of Internal Medicine, Yurin Hospital, Tokyo, Japan; 3 Department of Internal Medicine, Kawabata Hospital, Kyoto, Japan; 4 Department of Internal Medicine, Koyo Hospital, Wakayama, Japan

**Keywords:** Eosinophilic gastrointestinal disorders, Cytomegalovirus, Thiopurine

## Abstract

**Background:**

Eosinophilic enteritis is a chronic inflammatory disorder of the intestinal tract that is characterized by eosinophil infiltration. Cytomegalovirus (CMV), a common virus, has a broad infectivity range. CMV is retained in the host body after infection. Impairment of host immune defences may reactivate the latent CMV, leading to symptoms of overt disease.

**Case presentation:**

A Japanese female in her 70 s was admitted to a hospital due to diarrhoea and then transferred to our hospital. Laboratory data showed hypoalbuminemia. Computed tomography (CT) revealed oedema of the small intestine. Lower gastrointestinal endoscopy revealed oedema of the submucosa, without any remarkable changes in the mucosa of the terminal ileum. Histological examination of the terminal ileum revealed infiltration of > 20 eosinophils per high-power field (HPF). These findings aided in diagnosing eosinophilic enteritis. We administered methylprednisolone (500 mg/day) for three days, followed by tapering prednisolone. However, the patient’s general condition and hypoalbuminemia failed to improve. Immunoglobulin (Ig) G- CMV and IgM-CMV tests were positive. CMV antigenemia was extremely high. Therefore, we administered ganciclovir intravenously, which improved the patient’s condition. Furthermore, azathioprine was administered to taper and discontinue prednisolone without relapse of eosinophilic enteritis. This treatment helped stabilize the patient’s condition for approximately four years.

**Conclusion:**

We present a case of eosinophilic enteritis accompanied by CMV disease during prednisolone treatment. The patient’s condition improved after administration of ganciclovir. Azathioprine aided in discontinuing prednisolone and stabilizing the patient’s condition for approximately four years.

## Background

Eosinophilic enteritis is a chronic inflammatory disorder of the intestinal tract that is characterized by eosinophil infiltration [1]. Anti-inflammatory steroid drugs are used to treat eosinophilic enteritis, especially for patients in whom dietary restrictions are not feasible [2]. Thiopurines (azathioprine or 6-mercaptopurine) can be used [3].

Cytomegalovirus (CMV) is a common virus with a broad infectivity range. After infection, CMV is retained in the host body. When host immune defences are impaired, latent CMV is reactivated, leading to symptoms of overt disease [1].

Here, we present a case of eosinophilic enteritis accompanied by CMV disease during the treatment of eosinophilic enteritis. Treatment for CMV improved the patient’s symptoms. To avoid relapse of eosinophilic enteritis during steroid tapering, azathioprine was administered. After discontinuation of the steroid, azathioprine helped to maintain a prolonged stable status.

## Case presentation

**Fig. 1:**
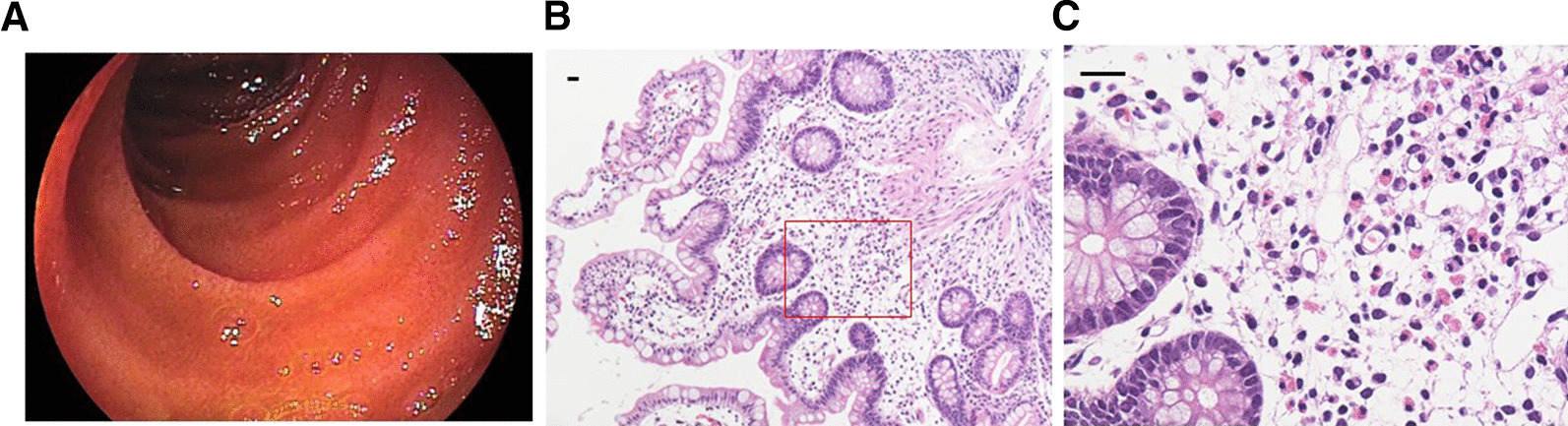
**A** Lower gastrointestinal endoscopy. Lower gastrointestinal endoscopy revealed oedema of the submucosa without any remarkable change in the mucosa in the terminal ileum. **B** Hematoxylin and eosin staining of the terminal ileum, low-power field. Scale bar, 20 µm. **C** Hematoxylin and eosin staining of the terminal ileum, high-power field (HPF). The histological examination showed the infiltration of > 20 eosinophils per HPF. Scale bar, 20 µm

A Japanese female in her 70 s was hospitalized due to diarrhoea. She had a history of Basedow’s disease and had undergone appendectomy for appendicitis. The patient did not have any pets. Three weeks before being transferred to our hospital, she experienced diarrhoea, abdominal pain, and vomiting. The laboratory data at the previous hospital showed a white blood cell count of 5,800 cells/μL and albumin level of 3.5 g/dL. After three weeks, the white blood cell count increased to 9,200 cells/μL and albumin level decreased to 1.3 g/dL. Eosinophils were not observed in the blood. Computed tomography (CT) indicated oedema of the small intestine. Lower gastrointestinal endoscopy revealed oedema of the submucosa, without any remarkable changes in the mucosa of the terminal ileum. Lower gastrointestinal endoscopy (EG-300MP, FUJIFILM, Tokyo, Japan) did not detect signs of ulcerative colitis or Crohn’s disease (Fig. 1A). Colonoscopy indicated oedema of the submucosa without any remarkable change in the mucosa of the whole colon and rectum. Upper gastrointestinal endoscopy did not detect any remarkable findings in the stomach, but identified redness in the duodenum. Thus, eosinophilic enteritis, lupus enteritis, and parasitic infections were suspected. Biopsies were performed using upper and lower gastrointestinal endoscopy. Total parenteral nutrition was administered. The levels of anti-nuclear antibody, anti-DNA antibody, anti-double strand DNA antibody, and lupus antibody were not elevated (Table 1). The results of faecal microbial ova-parasite inspection were negative. There was no evidence of lupus enteritis or parasitic infections; thus, eosinophilic enteritis was suspected. Furthermore, prednisolone (40 mg/day) was intravenously administred; however, no improvement was observed. Therefore, the patient was transferred to our hospital.

**Table 1:** Laboratory data

<Count of blood cells>		<Biochemistry>						
WBC	21,250	/μl	T-bil	0.6	mg/dL	Na	131	mEq/L
RBC	428	X104/μl	AST	45	IU/L	K	5	mEq/L
Hb	12.8	g/dl	ALT	56	IU/L	Cl	98	mEq/L
Ht	38.5	%	ALP	192	IU/L	Ca	7.3	mg/dL
Plt	31.6	X104/μl	γ-GTP	100	IU/L	CRP	0.02	mg/dL
Neutro	93.2	%	LDH	224	IU/L	HbA1c	5.8	%
Eos	0	%	TP	2.8	g/dL			
Lymp	5.5	%	Alb	1.8	g/dL			
			Ch-E	41	IU/L			
<Coagulation ability>		AMY	IU/L	52				
PT	76.5	%	P-AMY	41	IU/L			
APTT	35.2	sec	Cre	0.56	mg/dL			
ESR	1	mm	BUN	25.6	mg/dL			
<Autoantibody>			<Thyroid>					
Rhuematoid factor	1	U/ml	TSH	7.42	μIU/ml			
Antinuclear antibody	< 40		FT3	2.48	pg/ml			
Anti-DNA antibody	2	IU/ml	FT4	0.94	ng/dl			
Anti-dsDNA IgG	< 1.2	IU/ml						
Anti-SS-A/RO antibody	< 1.0	U/ml	<Others>					
Anti-SS-B/LA antibody	< 1.0	U/ml	IgG	173	mg/dL			
Lupus antibody	0.9		IgA	62	mg/dL			
Anti-cardiolipin antibody	< 8	U/ml	IgM	14	mg/dL			
MMP-3	133.8	ng/ml	IgE	13	IU/ml			
Anti-SM antibody	1	U/ml	C3	45	mg/dL			
Anti-SCL-70 antibody	< 1.0	U/ml	C4	10	mg/dL			
Anti-centromere antibody	< 2.0		CH50	33	U/ml			
PR3-ANCA	(–)	IU/ml	IL-2	734	U/ml			
MPO-ANCA	(–)	IU/ml	Insect egg (egg collection method)	(–)				

**Fig. 2:**
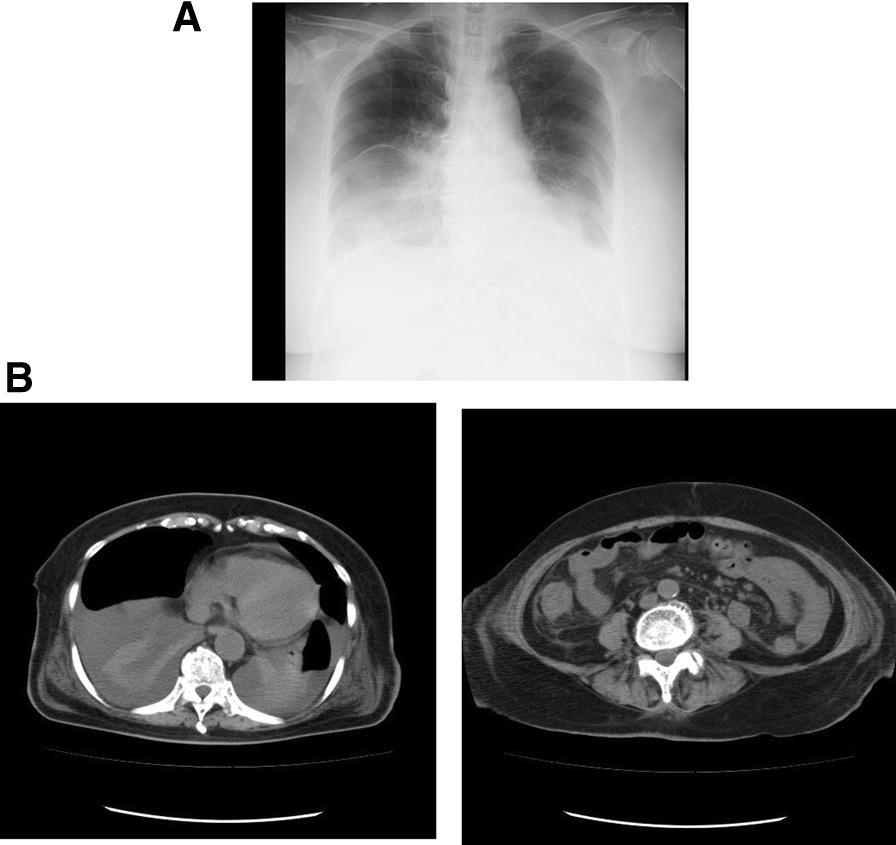
**A** Chest radiography. Chest radiography revealed massive pleural effusion. **B** Plain computed tomography (CT) upon transfer to our hospital. CT revealed massive pleural effusion, some ascites, and the thickening of the wall of the small intestine

The patient was suffering from seven instances of diarrhoea per day. The body was oedematous. The patient found it difficult to walk due to general fatigue. The patient’s body weight was 62 kg and her height was 142 cm. The patient’s body mass index was 31 kg/m^2^. Laboratory data showed increased white blood cell counts and hypoalbuminemia. Eosinophils were not observed in the blood (Table 1). The stool culture tested negative for pathogenic microorganisms. The results of faecal *Clostridium difficile* test were negative. The results of faecal microbial ova-parasite inspection were negative at our hospital. T-SPOT results were negative. There was no evidence of lupus enteritis or parasitic infection. Chest radiography revealed a massive pleural effusion (Fig. 2A). Sampling and analysis of the pleural effusion revealed a transudative pleural effusion (Table 2). CT revealed a massive pleural effusion, some ascites, and thickening of the wall of the small intestine (Fig. 2B). The patient’s general condition was critical for us to perform examination of the small intestine. We obtained the biopsy results from the previous hospital. Histological examination (ECLIPSE 80i, Nikon, Tokyo, Japan) of the terminal ileum revealed infiltration of > 20 eosinophils per high-power field (HPF). Epitheloid granuloma, virus inclusion bodies, amyloid deposition, or microorganisms, such as amoeba, were not observed (Fig. 1B and C). Biopsies of the ascending, transverse and sigmoid colon and rectum failed to show eosinophilic infiltration. A biopsy of the stomach and duodenum failed to identify any remarkable findings. Diarrhoea, biopsy of the terminal ileum, and thickening of the wall of the small intestine led to a diagnosis of eosinophilic enteritis [4]. Four days after transfer to our hospital, we administered methylprednisolone (500 mg/day) intravenously for three days, and then reduced the prednisolone dose.

**Table 2:** Pleural effusion

Cell count	104
Neutrophil	1%
Eosinophil	0%
Basophil	0%
Lymphocyte	82%
Monocyte	0%
Mesothelial cells	5%
Macrophage	12%
Total protein	50% g/dL
Sugar	269 mg/dL
Amylase	60 IU/mL
LDH	63 IU/L

Thirteen days after transfer to our hospital, the frequency of diarrhoea decreased gradually to twice a day. However, the hypoalbuminemia failed to improve. Ten days after transfer to our hospital, the patient developed a fever of 38 ℃. The C-reactive protein (CRP) levels increased gradually, and the platelet count decreased. Urinary tract infection was diagnosed, and antibiotics were administered. Fourteen days after transfer to our hospital, the lactate dehydrogenase level was elevated to 327 IU/L. Immunoglobulin (Ig) G- CMV and IgM-CMV tested positive. CMV antigenemia (C7-HRP) levels were measured. In 150,000 white blood cells, 222 positive cells were detected, and in another 150,000 white blood cells, 192 positive cells were detected. These numbers were much higher than the threshold of 1. Therefore, we considered it necessary to treat the CMV disease and intravenously administer ganciclovir at a dose of 200 mg twice a day for two weeks. After the administration of ganciclovir, the CRP levels decreased, and CMV antigenemia decreased to < 10 positive cells out of 150,000 white blood cells.

**Fig. 3:**
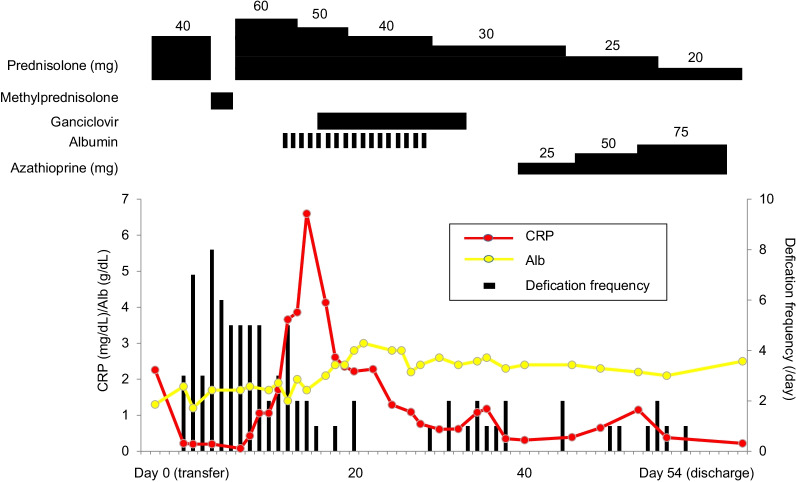
Clinical course

**Fig. 4:**
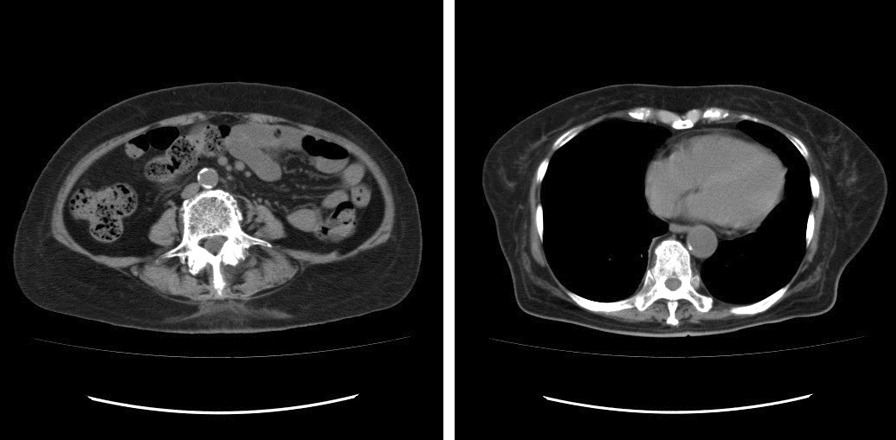
Plain computed tomography (CT) on discharge. CT revealed decreased pleural effusion and ascites and improved of wall thickening of the small intestine

Twenty-two and 37 days after the transfer, we administered montelukast and epinastine, respectively. The patient experienced delirium; therefore, we considered it necessary to taper prednisolone. Thirty-eight days after the transfer, we administered azathioprine while tapering the prednisolone dose. One month after the transfer, ordinary stool was observed, the pleural effusion had decreased, a diet was initiated, and total parenteral nutrition was discontinued. The patient’s oedema improved, but albumin levels failed to increase despite intravenous administration (Fig. 3). CT revealed a decrease in pleural effusion and ascites, and an improvement in wall thickening of the small intestine (Fig. 4). The patient was discharged 54 days after transfer to our hospital. The body weight decreased from 62 kg on admission to 50 kg on hospital discharge. The albumin level gradually increased to > 3.5 g/dL two months after discharge. The prednisolone dose was reduced, and then discontinued seven months after discharge. After discontinuation of prednisolone, the white blood cell count decreased. We decreased the azathioprine dose to 25 mg once every two days. Approximately four years after discharge, the patient was healthy, with albumin level > 3.8 g/dL. The eosinophilic enteritis had not been relapsed at the time of drafting of this report.

## Discussion and conclusion

This report describes a case of eosinophilic enteritis accompanied by CMV disease during treatment. Treatment with CMV improved the patient’s status. To safely discontinue steroids, azathioprine was administered and helped maintain a stable health status.

We initially considered that other allergic diseases, Wegener’s granulomatosis (granulomatosis with polyangiitis), *Aspergillus* and other fungal infections, hypothyroidism, and common variable immune deficiency should be confirmed. The patient did not have any other allergic disease. We perceived that it was difficult to diagnose Wegener’s granulomatosis (granulomatosis with polyangiitis). There are three reasons for this. First, kidney disorders were not observed. Second, PR3-ANCA was negative. Third, CT scans failed to detect nodules, infiltration, cavities, or ground-glass attenuation/opacity in the lung, or sinusitis. *Aspergillus* antigen and β-D-glucan were negative throughout the course of treatment. A CT scan failed to detect *Aspergillus* or other fungal infections. These results failed to indicate the possibility of infection. The patient had a history of Basedow’s disease. On transfer to our hospital, the levels of TSH, FT3, and FT4 were 7.42 μIU/ml, 2.48 pg/ml, and 0.94 ng/dl, respectively. We negated the hypothesis that only hypothyroidism caused the disease. Elven months later, the data showed euthyroidism. The patient’s presentation was atypical for common variable immune deficiency, due to the absence of a history of sinusitis or repeated pneumonia, and due to the absence of sinusitis, splenomegaly, hepatomegaly, lymphadenopathy, or thymoma in the CT scan.

Hypoalbuminemia was considered to be related to severe diarrhoea and decreased food intake because the albumin levels were not low on admission to the previous hospital. The pleural effusion was transudative. Thus, we hypothesized that the pleural effusion was due to hypoalbuminemia.

Anti-inflammatory steroid drugs have been used to treat eosinophilic enteritis. This is the main therapy used for patients in whom dietary restrictions are not feasible [2]. Steroids were administered using a semipulse protocol. In the present case, prednisolone (40 mg/day) was administred intravenously at the previous hospital; however, diarrhoea, oedema, and hypoalbuminemia failed to improve. Thus, we hypothesized that a regular dose of steroid was insufficient for the disease control and that the disease was steroid refractory. There is no established steroid usage for eosinophilic enteritis. There are a few case studies in which steroid pulse therapy was administred for eosinophilic gastroenteritis [5, 6]. Therefore, based on these findings, we administred a steroid semipulse.

CMV remains latent in most humans, but it may become activated and the patient may develop symptoms of overt disease, especially when the host immune defences are impaired [1, 7]. Takizawa et al. reported that out of 7,377 patients with rheumatic disease, 151 were diagnosed with CMV infection; and in addition to oral corticosteroids for all, except one patient, 81 were treated with pulsed methylprednisolone, 64 with cyclophosphamide, and 36 with other immunosuppressants [8]. Fourteen days after transfer to our hospital, IgG-CMV and IgM-CMV tested positive. It has been reported that out of 43 cases of CMV colitis in patients without inflammatory bowel disease, two showed macroscopically normal endoscopic findings [9]. Therefore, it was difficult to identify whether CMV reactivated before or after steroid administration. After admission to our hospital, the platelet count remained high, but the lactate dehydrogenase level was not elevated. After semipulse steroid administration, the platelet count decreased, and the lactate dehydrogenase level increased. Thus, we thought that CMV was reactivated after steroid semipulse treatment. CMV antigenemia was extremely high. The sensitivity and specificity of antigenemia for CMV gastrointestinal disease are reported to be 65.4%, and 93.6%, respectively [10]. Mean peak antigenemia levels are significantly higher in patients with symptomatic CMV disease than in those with asymptomatic CMV disease [11]. Based on this, we concluded that ganciclovir should be administered. Improvement was achieved after administering ganciclovir. Takeyama et al. reported eosinophilic gastroenteritis with CMV infection in a three-year-old male [1]. However, to the best of our knowledge, this is the first report of CMV disease that developed during the treatment of eosinophilic enteritis in an adult.

Steroid-dependent or steroid-refractory patients may be treated with thiopurines (azathioprine or 6-mercaptopurine). Redondo-Cerezo et al. reported a case in which treatment with azathioprine helped maintain remission of eosinophilic gastroenteritis after steroid discontinuation due to depressive symptoms [12]. Netzer et al. reported a case in which treatment with azathioprine induced and maintained long-term remission of corticosteroid-dependent eosinophilic gastroenteritis concomitant with eosinophilic oesophagitis (range, 3–8 years) [13]. Azathioprine inhibits the proliferation of T and B lymphocytes, which decreases the production of cytotoxic T lymphocytes and plasma cells. In eosinophilic oesophagitis, increased oesophageal infiltration with cluster of differentiation (CD)3^+^ T cells, CD8^+^ T cells, CD1a^+^ dendritic cells, and mast cells has been reported [14, 15]. Netzer et al. postulated that azathioprine delays the recruitment and/or proliferation of lymphocytes in the oesophageal epithelium, leading to decreased antigen processing in the oesophagus [13]. Copeland et al. reported the possibility that the levels of interleukin (IL)-3, granulocyte macrophage colony-stimulating factor, IL-5, and eotaxin are downregulated or inhibited by the use of immunosuppressive mediators in eosinophilic gastritis [16]. In the present case, the disease was steroid-refractory. Moreover, azathioprine was administered during steroid tapering, and remission was achieved for approximately three years after azathioprine administration. To the best of our knowledge, this is the first report of such a long use of azathioprine.

In conclusion, this report presents a case of eosinophilic enteritis accompanied by CMV disease during steroid treatment of eosinophilic enteritis. The patient’s general condition improved after administration of ganciclovir. Azathioprine helped maintain a stable status after the tapering and discontinuation of prednisolone for approximately four years.

## Data Availability

Not applicable.

## Abbreviations

CMV: Cytomegalovirus
CT: Computed tomography
HPF: High-power fields
Ig: Immunoglobulin
CRP: C-reactive protein
CD: Cluster of differentiation
IL: Interleukin
